# Resection extent and BRAF V600E mutation status determine postoperative tumor growth velocity in pediatric low-grade glioma: results from a single-center cohort analysis

**DOI:** 10.1007/s11060-022-04176-4

**Published:** 2022-11-01

**Authors:** David Gorodezki, Julian Zipfel, Manon Queudeville, Jordana Sosa, Ursula Holzer, Jan Kern, Andrea Bevot, Jens Schittenhelm, Thomas Nägele, Martin Ebinger, Martin U. Schuhmann

**Affiliations:** 1 Department of Hematology and Oncology, University Children’s Hospital Tübingen, Tübingen, Germany; 2 Section of Pediatric Neurosurgery, Department of Neurosurgery, University Hospital Tübingen, Tübingen, Germany; 3 Department of Neuropediatrics and Developmental Neurology, University Hospital Tübingen, Tübingen, Germany; 4 Department of Neuropathology, Institute of Pathology, University Hospital Tübingen, Tübingen, Germany; 5 Department of Neuroradiology, University Hospital Tübingen, Tübingen, Germany; 6 Clinic for Pediatric Hematology and Oncology, University Medical Center Hamburg-Eppendorf, Hamburg, Germany

**Keywords:** Low-grade glioma, Surgery, Senescence, BRAF V600E

## Abstract

**Purpose:**

Despite excellent long-term overall survival rates, pediatric low-grade gliomas (pLGG) show high variety of clinical behavior regarding progress or senescence post incomplete resection (IR). This study retrospectively analyzes tumor growth velocity (TGV) of pLGG before surgery and after IR to investigate the impact of surgical extent, tumor location and molecular BRAF status on postoperative residual tumor growth behavior.

**Methods:**

Of a total of 172 patients with pLGG receiving surgical treatment, 107 underwent IR (66%). Fifty-three vs 94 patients could be included in the pre- and post-operative cohort, respectively, and were observed over a mean follow-up time of 40.2 vs 60.1 months. Sequential three-dimensional MRI-based tumor volumetry of a total of 407 MRI scans was performed to calculate pre- and postoperative TGV.

**Results:**

Mean preoperative TGV of 0.264 cm^3^/month showed significant deceleration of tumor growth to 0.085 cm^3^/month, 0.024 cm^3^/month and −0.016 cm^3^/month after 1st, 2nd, and 3rd IR, respectively (p < 0.001). Results remained significant after excluding patients undergoing (neo)adjuvant treatment. Resection extent showed correlation with postoperative reduction of TGV (R = 0.97, p < 0.001). ROC analysis identified a residual cut-off tumor volume > 2.03 cm^3^ associated with a higher risk of progress post IR (sensitivity 78,6%, specificity 76.3%, AUC 0.88). Postoperative TGV of BRAF V600E-mutant LGG was significantly higher than of BRAF wild-type LGG (0.123 cm^3^/month vs. 0.016 cm^3^/month, p = 0.047).

**Conclusion:**

This data suggests that extensive surgical resection may impact pediatric LGG growth kinetics post incomplete resection by inducing a significant deceleration of tumor growth. BRAF-V600E mutation may be a risk factor for higher postoperative TGV.

**Supplementary Information:**

The online version contains supplementary material available at 10.1007/s11060-022-04176-4.

## Introduction

Pediatric low-grade glioma (pLGG) comprise the most common CNS tumors of childhood, accounting for 30–50% of primary brain tumors in children and adolescents [1]. Despite excellent long-term overall survival rates (OS) following various degree of resection, survivors of pLGG are prone to suffer from functional, neurologic, and endocrine complications from their disease or treatment. Therapeutic strategies are challenged to provide maximum tumor control while minimizing irreversible long-term functional impairment [2–4].

Compared to LGG in adults, pLGG shows high variety of clinical behavior and limited predictability in terms of progress or senescence of tumor residuals, as prospective studies have reported 5-year PFS of 45% to 65% after incomplete resection (IR). Although the majority of LGG show indolent growth rates and spontaneous regression has been observed, late progression after years of senescence post primary therapy has been reported [3, 6, 7, 10–12]. Malignant transformation occurs scarcely in childhood LGG [13, 14].

As population-based cohort studies have identified the extent of surgery as the predominant factor for favorable OS and PFS, surgery remains the crucial element among advancing therapeutic approaches [2, 3, 5–10]. However, resectability can be severely limited by surrounding eloquent anatomical structures, as population-based cohort studies show consistently high rates of IR in 65–73% of cases [2, 5–7].

As molecular profiling studies have incrementally identified key genetic alterations in pLGG affecting the RAS/MAPK pathway over the last decade, most somatic events appear to include BRAF alterations. [15, 16]. While the most frequent molecular alteration in pLGG (KIAA1549-BRAF fusion) is currently suspected to have a favorable impact on OS and PFS, pediatric LGG harboring the most common mutation in BRAF (BRAF V600E) is presumably associated with unfavorable PFS [17–19]. By contrast, in vitro studies on pilocytic astrocytoma cells with BRAF alterations showed a tendency to senescence after an initial period of growth, suggesting that a subgroup of pLGG may exhibit growth deceleration over time [20, 21]. However, the prognostic role of these aberrations is currently discussed and remains unclear [15–23].

In this study, we analyzed the tumor growth velocity (TGV) of pLGG to investigate the impact of resection extent, tumor location and the most frequent BRAF aberrations on postoperative growth following IR. Therefore, using residual tumor volumetry to calculate tumor growth velocity, we compared tumor kinetics in sequential MRI follow-up examinations before and after surgical resection in a retrospective approach, aiming to identify potential variables to prognosticate further progress or senescence post subtotal resection.

## Patients and methods

### Selection criteria

We searched the database of the Children's University Hospital of Tübingen for patients at the age of < 18 years at time of diagnosis, treated with LGG CNS WHO grade I or II according to the 2021 WHO classification of tumors at all CNS sites between 2006 and 2020 [24].

Inclusion criteria for this study were the availability of a minimum of two sequential MRI scans with defined sequences over a period no shorter than 6 months both before surgery (preoperative cohort) and after IR (postoperative cohort). Patients with radiographically confirmed GTR and no signs of relapse during the observation period were excluded from the postoperative cohort.

### Methods

Patient data concerning tumor site, resection status and (neo)adjuvant treatment were obtained from the hospital database. Histopathological diagnoses, BRAF-KIAA1549 fusion status and BRAF-V600E mutation status were obtained from institutional and central pathology reports (German Reference Center for Paediatric Brain Tumors). Testing for BRAF V600E-mutation was performed via pyrosequencing following PCR amplification of the BRAF gene from extracted tumor DNA.

For patients with hypothalamic chiasmatic tumors associated with NF-1, radiological diagnosis of pilocytic astrocytoma was accepted, as biopsy has shown to be redundant in this constellation [25]. Radiological diagnosis of LGG was furthermore accepted in tree non-NF-1 patients with characteristic imaging features of optic pathway glioma and no current indication for treatment.

MRI-based volumetry of pre- and postoperative tumor burden was serially performed on sequential axial MRI scans. Most images were taken at 1.5 T MRI with 1–3 mm slices. Three-dimensional tumor volumetry was performed using BrainLab Elements (version 3.0, BrainLab, Munich, Germany). Repeated volumetries of various investigators showed negligible variation of tumor volume measurements. A total of 407 MRI scans were analyzed, corresponding to 3.1 MRI scans per eligible patient.

### Statistical analyses

Most analyses were performed using JMP 15.2.0 (SAS Institute Inc., Cary, North Carolina, USA). Anderson–Darling test was applied to analyze distribution of pre- and postoperative tumor growth rates. Due to not normally distributed data, nonparametric testing was performed for further analysis using Kruskal–Wallis test and Mann–Whitney rank sum test. Linear regression analysis, log rank test, one sample t-test and ROC analysis were performed using GraphPad Prism 8.0 (GraphPad Software, Inc., California, USA). Statistical significance was defined at p values ≤ 0.05.

## Results

A total of 191 patients were treated at our institution for pLGG between 2006 and 2020. Diagnoses included pilocytic astrocytoma °1 (137), ganglioglioma °1 (36), astrocytoma °2 (14), oligodendroglioma °2 (1), pleomorphic xanthoastrocytoma °1 (1), rosette-forming glioneural tumor (RGNT) °1 (1) and subependymal giant cell astrocytoma (SEGA) °1 (1). Tumor sites included the posterior fossa (80), supratentorial midline and optic nerve (55), cerebral hemispheres (46), spinal cord (8) and lateral ventricles (2). Overall, 172/191 patients (90.1%) underwent surgical therapy. GTR was achieved in 65 cases (38%), of whom 5 patients (7.7%) showed recurrence during follow-up. 107 patients (62%) received IR, defined as visible tumor remnants described by institutional and reference neuroradiology reports.

Ninety-four patients with tumor residuals could be included into in postoperative cohort, 53 patients met eligibility criteria for the preoperative cohort. Mean follow-up period of the pre- and postoperative cohort was 40.2 ± 36.1 and 60.1 ± 42.3 months, respectively.

Within the postoperative cohort, 51 patients (54%) fulfilled definition of subtotal resection (> 90% resection of initial tumor volume, STR). Partial resection (classified as < 90% resection of initial tumor volume, PR) was achieved in 43 patients (46%). STR was achieved in 30 (65%) of tumors located in the posterior fossa, 16 (76%) tumors located in the cerebral hemispheres and 5 (29%) of supratentorial midline gliomas.

Fifty-five (59%) patients showed no significant tumor growth following IR, including 36 (71%) of subtotally resected tumors and 19 (44%) of partially resected tumors.

**Fig. 1:**
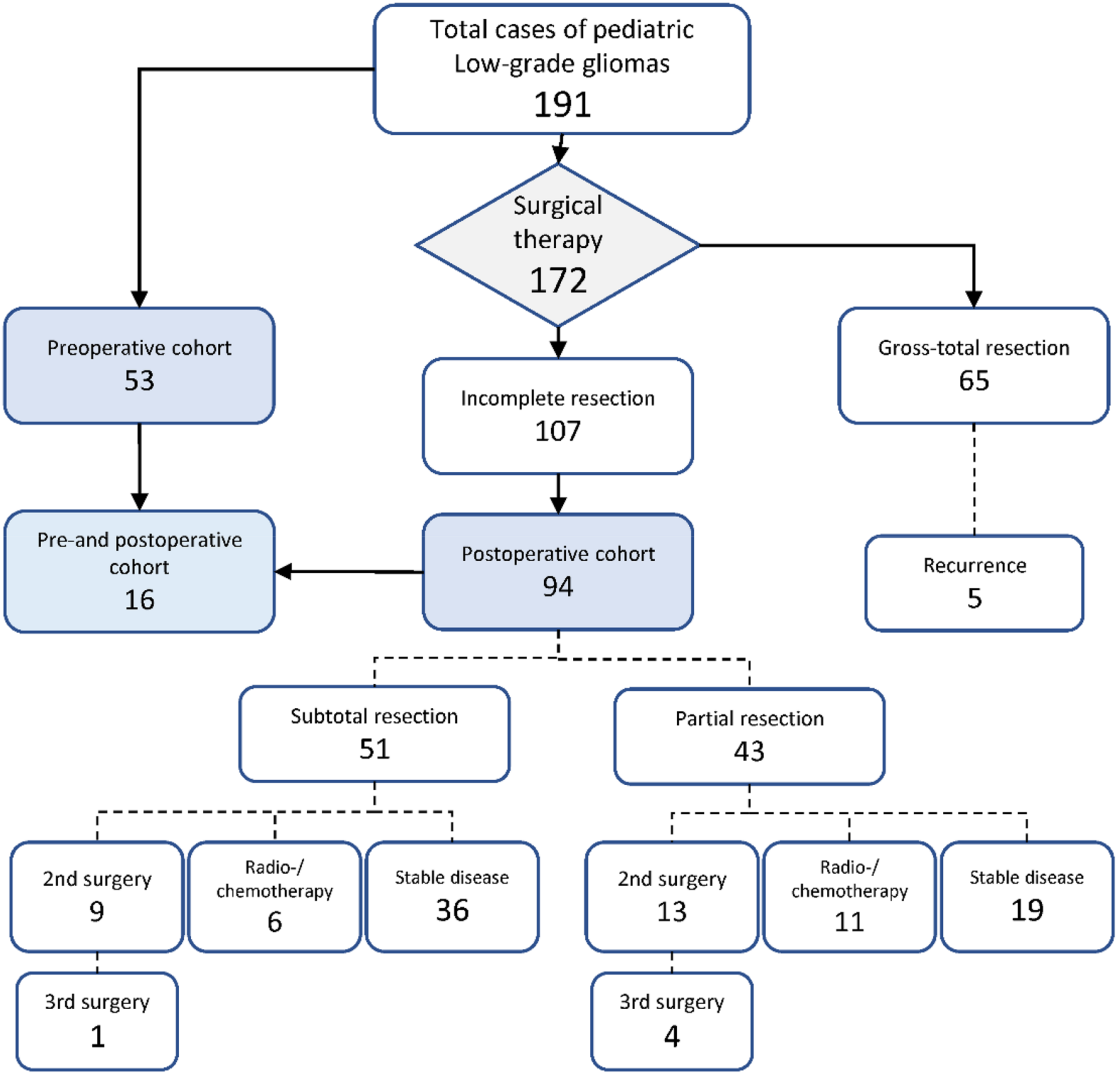
Distribution of cases within the analyzed single-center cohort of 191 patients treated with pediatric LGG at our institution between 2006 and 2020

Within the postoperative cohort, 22 patients (23%) after IR underwent second surgery following significant growth of residual tumor remnants within a mean follow-up period of 25 ± 17 months. Twenty patients (21%) post IR received further oncological treatment other than surgery due to progress and limited surgical options. A third intervention was necessary in 5 patients (5.3%) after a further mean FU of 21 ± 10 months. Distribution of cases in a CONSORT flow diagram is illustrated in Fig. 1.

### Comparison of pre- and postoperative tumor growth velocity (TGV)

**Fig. 2:**
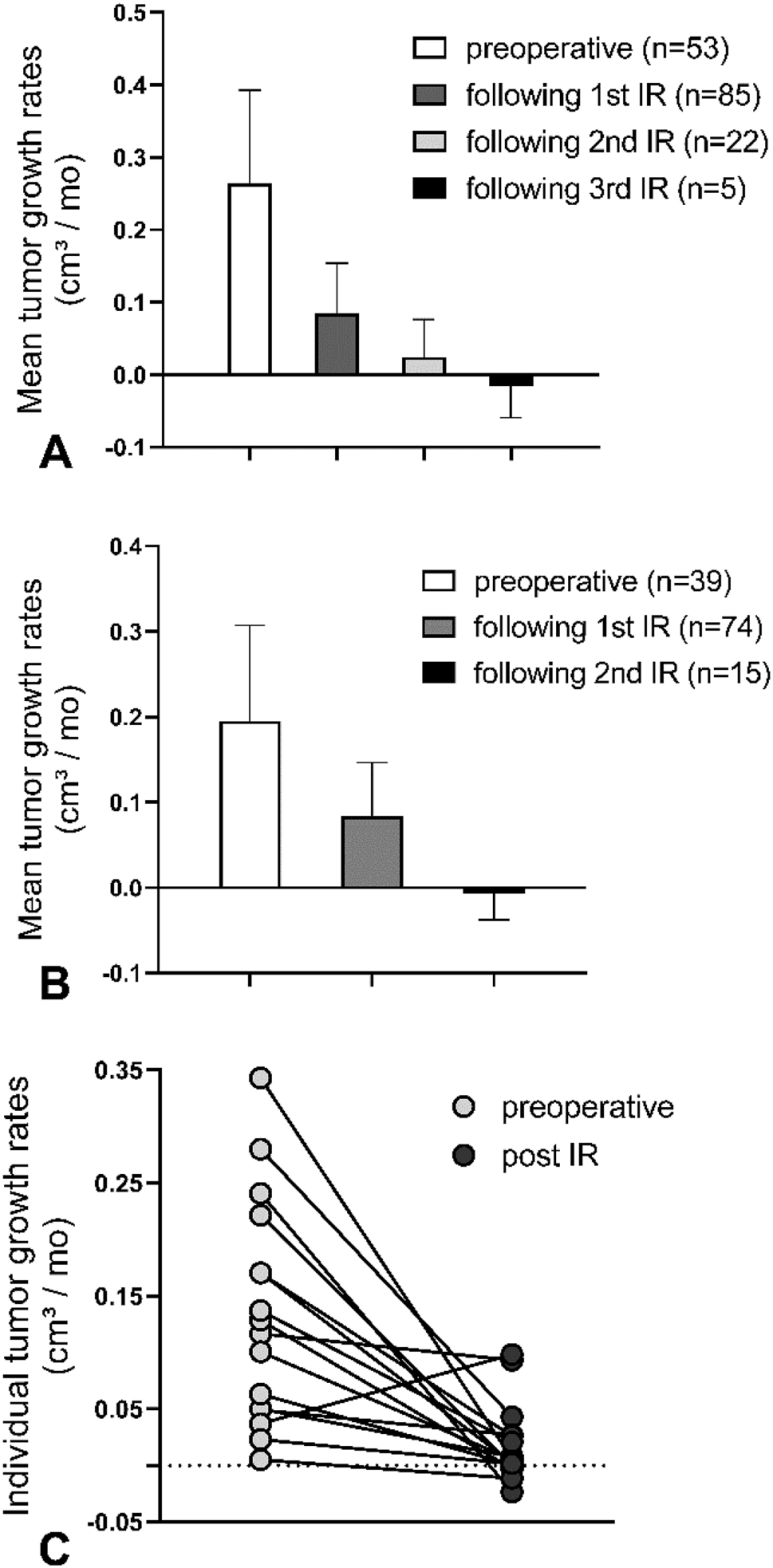
**A** Comparison of pre- and postoperative mean tumor growth rates showed a significant deceleration of tumor growth after 1st, 2nd and 3rd IR (Kruskal–Wallis test, p = 0.001, bars show mean and 95% CI). Pairwise Mann–Whitney tests showed significant difference of mean preoperative growth velocity and mean growth rate following 1st IR (p < 0.02), 2nd IR (p = 0.015) and 3rd IR (p < 0.001). **B** Results remained significant after excluding patients undergoing (neo)adjuvant treatment (Kruskal–Wallis test, p = 0.037, bars show mean and 95% CI). **C** Intra-patient comparison of individual tumor growth rates before and after IR. Tumor growth rates decreased by an average of 84.7% (p = 0.0024)

Comparative analysis of pre- and postoperative TGV showed a mean preoperative growth velocity of 0.264 cm^3^/month (*n* = *53*), while postoperative TGV after first IR accounted for 0.085 cm^3^/month (*n* = *85*). After eliminating 46/85 patients (54%) with no measurable postoperative growth of residual tumor from the cohort, postoperative TGV after first STR accounted for 0.112 cm^3^/month (*n* = *39*). Postoperative TGV after second and third STR showed a mean of 0.024 cm^3^/month (*n* = *22*) and -0.016 cm^3^/month (*n* = *5*), respectively (see Fig. 2A). Tumor regression in 3/5 cases after third STR resulted in a slightly negative mean TGV. Data sets showed non-standard distribution and variate analysis showed a significant difference (Kruskal–Wallis test, p = 0.001). Pairwise Mann–Whitney tests showed significant difference of mean preoperative TGV and mean growth rate following 1st STR (p = 0.02), 2nd STR (p = 0.015) and 3^rd^ STR (p < 0.001).

To investigate solely the impact of surgery on postoperative TGV, for all subsequent analyses patients who had obtained neoadjuvant oncological treatment (chemotherapy, radiotherapy, or targeted therapy) were further on excluded from both cohorts, and radiological follow-up post IR was ended when adjuvant treatment was administered. Comparison of pre- and postoperative tumor growth rates showed a mean preoperative TGV of 0.195 cm^3^/month (n = 39). Postoperative TGV showed an average of 0.038 cm^3^/month after first surgery (n = 74) and -0.007 cm^3^/month (n = 15) after second surgery (Fig. 2B). Variate analysis showed a significant difference (Kruskal–Wallis test, p = 0.037).

For a subset of 12 patients receiving surgery as the only oncological treatment, observation both before and after STR accounted for a minimum of 6 months respectively, allowing intra-patient comparison of pre- and postoperative TGV. Individual differences in TGV before and after STR are comparatively shown in Fig. 2C. Tumor growth rates decreased by an average of 84.7% (p = 0.0024) Increase of TGV after STR has been observed in one case.

### Impact of resection extent on postoperative tumor growth velocity (TGV)

**Fig. 3:**
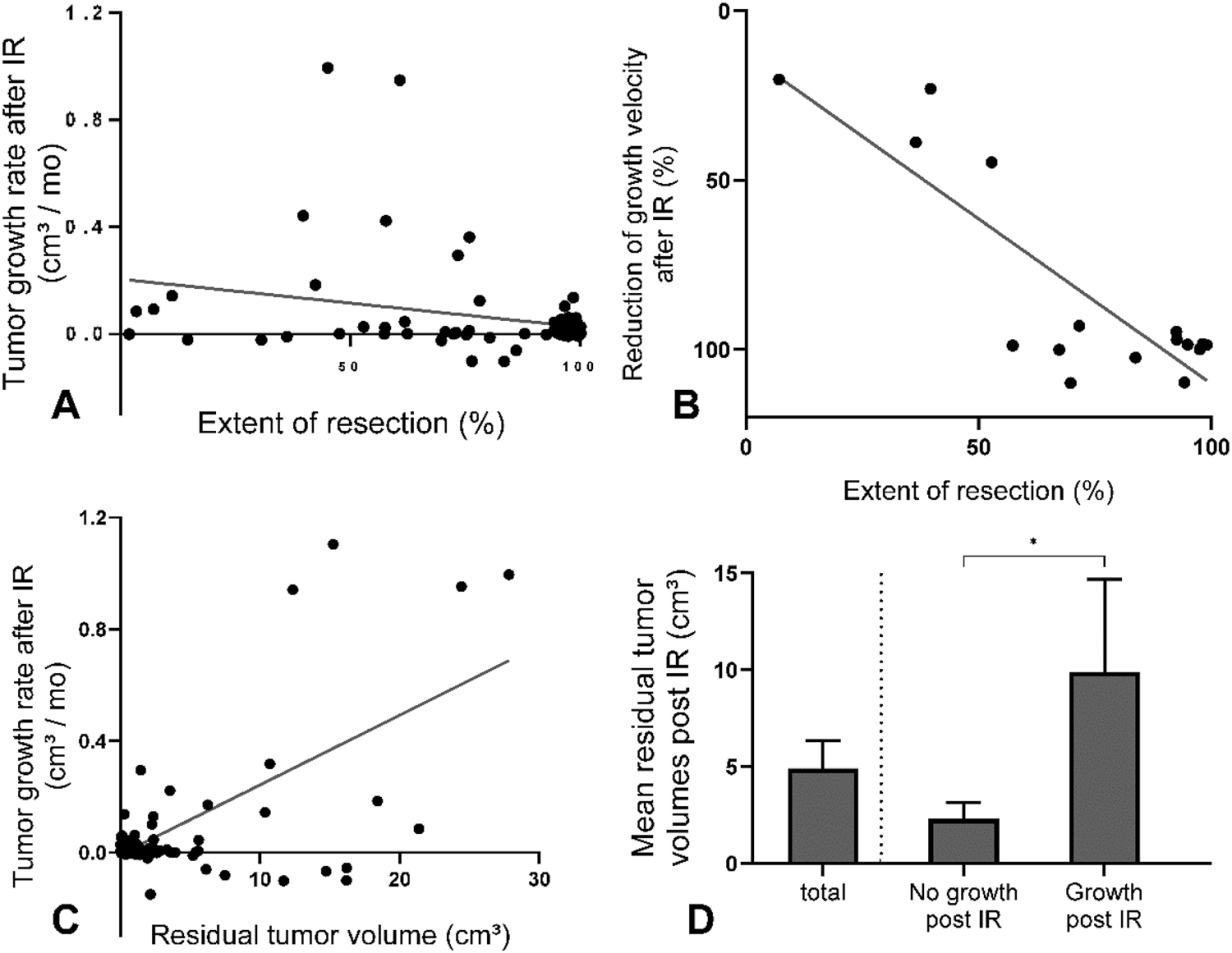
**A** Linear regression including 71 cases of incomplete resection showed a minor, thus significant negative correlation between extent of resection and postoperative growth rates (R = − 0.02, R squared = 0.07, p = 0.027). **B** A significant correlation between resection extent (%) and percental reduction of growth velocity after STR could be shown in 16 patients not receiving (neo)adjuvant treatment (R = 0.974, R squared = 0.719, p < 0.001). **C** Linear regression analysis revealed a significant impact of residual tumor burden post incomplete resection on postoperative growth velocity (R = 0.025, R squared = 0.3, p < 0.001). **D** Comparing mean residual tumor volumes of cases with measurable tumor growth vs tumor residuals with no signs of growth during the observation period post IR showed a significant difference of mean tumor volumes (9.308 cm^3^ [n = 19] vs 2.308 cm^3^ [n = 55], p = 0.011)

Resection extent was quantified on basis of tumor burden in consecutive pre- and postoperative MRI scans. Linear regression showed a minor, but significant negative correlation between extent of resection and TGV after IR (n = 71, R = − 0.02, R squared = 0.07, p = 0.025), as shown in Fig. 3A.

**Fig. 4:**
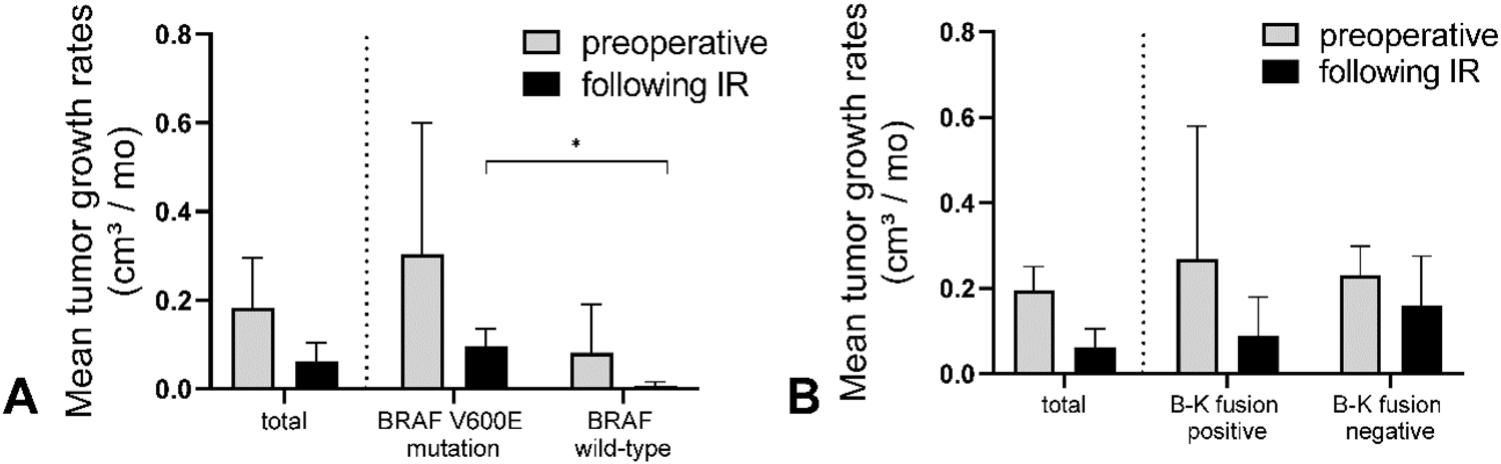
A *Comparison of postoperative growth velocities of BRAF V600E mutant LGG and BRAF wild-type LGG showed significant difference of means (*0.123 cm^3^/month and 0.016 cm^3^/month). **B** Comparative analysis of pre- and postoperative tumor growth rates in BRAF-KIAA1549 fusion positive and negative LGG showed no significant differences of means (p = 0.17 and p = 0.09, respectively)

Within the postoperative cohort, partially resected LGG showed a mean TGV of 0.113 cm^3^/month (n = 39), while mean postoperative TGV after subtotal resection (STR) was 0.047 cm^3^/month (n = 46). Mann–Whitney rank sum test showed a significant difference (p = 0.02).

For a subset of 16 patients not receiving any (neo)adjuvant treatment, follow-up period of > 6 months both prior and post STR allowed illustration of correlation between individual percental resection extent and relative decrease of individual tumor growth velocity compared to preoperative tumor growth rates. Linear regression showed significant correlation, as shown in Fig. 3B (R = 0.974, R squared 0.719, p < 0.001).

### Impact of residual tumor volume on postoperative growth velocity (TGV) and calculation of a cut-off tumor volume to predict potential progress post IR

A significant positive correlation could be shown between the amount of residual tumor volume and postoperative TGV by linear regression, as illustrated in Fig. 3C (n = 85, R = 0.025, R squared = 0.3, p < 0.001). Comparing mean residual tumor volumes of cases with radiologically measurable tumor growth vs tumor residuals with no signs of growth during the observation period post IR showed a significant difference of mean tumor volumes (9.308 cm^3^ vs 2.308 cm^3^, p = 0.011), see Fig. 3D.

We conducted a ROC analysis in order to identify a potential cut-off tumor value post IR associated with a higher risk of radiologically detectable tumor growth during the follow up period within the postoperative cohort. A feasable cut-off tumor value was found at 2.03 cm^3^ (sensitivity 78.6%, specificity 76.3%, AUROC curve 0.88, p < 0.001).

### Impact of tumor location on resection extend and pre- and postoperative tumor growth velocity

Furthermore, we compared pre- and postoperative TGV stratified by tumor location. Incomplete resection induced a significant deceleration of mean TGV in LGG located in the posterior fossa and the cerebral hemispheres (Mann–Whitney rank sum test, p = 0.025 and p = 0.018, respectively), the two main tumor locations in which the highest rates of STR could be achieved. However, significant impact of IR on TGV in supratentorial midline glioma could not be shown (p = 0.11). Results are listed in Table 1.

**Table 1:** Illustration of mean pre- and postoperative tumor growth rates ± SD distinguished by tumor location

Tumor location	Preoperative tumor growth rate	Postoperative tumor growth rate	Statistical significance
Posterior fossa (PF)	0.22 ± 0.2 n = 7	0.04 ± 0.102 n = 36	p = 0.025
Supratentorial midline (SMG)^a^	0.169 ± 0.472 n = 13	0.11 ± 0.261 n = 16	p = 0.11
Cerebral hemispheres (CH)	0.179 ± 0.212 n = 20	0.004 ± 0.019 n = 17	p = 0.018
Total	0.1847 ± 0.357 n = 38	0.057 ± 0.180 n = 80	p = 0.037

^a^Included: opticohypothalamic tumors and tumors of the basal ganglia

### Impact of BRAF V600E mutation and BRAF-KIAA1549 fusion on pre- and postoperative tumor growth velocity

We investigated the impact of BRAF V600E mutation status on pre- and postoperative growth rates. BRAF V600E mutation status was analyzed in 18/53 cases (34%) within the preoperative cohort during a later performed resection, and BRAF V600E mutation was detected in 8/18 cases (44%).

In postoperative cohort, BRAF V600E mutation status was tested in 55/94 cases (59%), and 12/55 (21%) cases were positive. In the preoperative cohort, BRAF V600E-mutated LGG showed a mean TGV of 0.305 cm^3^/month (n = 8), while BRAF wild-type glioma showed a mean TGV of 0.082 cm^3^/month (n = 10), while missing statistical significance (p = 0.09). Within the postoperative cohort, BRAF V600E-mutated glioma showed a mean TGV of 0.123 cm^3^/month (n = 12), while BRAF wild-type glioma showed a significantly lower mean TGV of 0.016 cm^3^/month (n = 43, p = 0.047). Results are illustrated in Fig. 4A.

BRAF-KIAA1549 (B-K) fusion was only detected in pilocytic astrocytoma and tumors histologically classified as diffuse astrocytoma. Among cases of pilocytic astrocytoma, B-K fusion was detected in 37/49 cases (75%) and showed the highest frequency in midline glioma (11/11, 100%).

B-K fusion negative and B-K fusion positive tumors showed a mean preoperative TGV of 0.23 cm^3^/month (n = 10) and 0.27 cm^3^/month (n = 3), respectively. Analyzing the postoperative cohort, B-K fusion negative tumors showed a mean TGV of 0.16 cm^3^/month (n = 16), while B–K fusion positive tumors showed a mean TGV of 0.09 cm^3^/month (n = 33). No statistical significance was found in either group. Results are illustrated in Fig. 4B.

## Discussion

Of 191 Patients treated with pLGG between 2006 and 2020 at our institution, a total of 53 vs 94 patients in the pre- and postoperative cohort, respectively, could be included in our analyses. Distribution of both histopathological diagnoses and tumor sites within the cohorts showed similarity to previously published population-based cohort studies [2, 5, 6].

Mean follow-up period within our cohorts of 60.1 vs 40.1 months outlasted the median time to progression of approximately 28 months in a previously published pLGG cohort [19].

To decode the ambivalent biological behavior of pLGG after IR most precisely, we determined TGV in sequentially performed volumetric analyses using a specialized neuronavigational software, which has shown favorable intra-rater variability in previous evaluation [26]. Three-dimensional volumetric measures on the base of planimetry were used due to superior sensitivity to change in growth tracking compared to linear measurements, as previously shown in vestibular schwannoma [27].

Comparative analysis of pre- and postoperative TGV showed a significant decrease of tumor growth after surgical intervention, as this effect showed continuity after second and third IR. As this data implies, relative extent of resection and residual tumor volume appear to have a significant impact on postoperative TGV. This tendency appeared even more visible and highly significant in intra-patient analysis within the subgroup of patients with available pre- and postoperative tumor growth rates.

Further stratification of cases towards subtotally resected and partially resected tumors showed significantly lower mean tumor growth rates post STR compared to PR. Remarkably, 39/51 (70%) of subtotally resected tumors vs 19/43 (44%) of partially resected tumors showed stable disease without any radiological indication of progression within the observation period, thus providing no indication for further local or systemic therapy, similar to cases post GTR with no sign of recurrence. Those ‘silent’ tumor residuals post IR showed a significantly lower mean tumor volume compared to pLGG with radiographically detectable tumor growth post IR. ROC analysis identified a residual cut-off tumor volume > 2.03 cm^3^ associated with a higher risk of radiologically detectable progress post IR.

The impact of tumor location on resection extent and thus postoperative tumor growth rates is well illustrated by the comparative analysis of pre- and postoperative TGV distinguished by tumor location. As the highest rates of STR could be achieved in pLGG located in the cerebral hemispheres and the posterior fossa (76% and 65%, respectively) compared to supratentorial midline (29%), a significant deceleration of tumor growth post IR could solely be shown in the first two subgroups. Lower rates of STR in tumors surrounded by highly eloquent brain tissue located in the supratentorial midline appears to be in line with previous publications, showing that extensive resection of pLGG in deep-seated midline locations appears rarely possible, often associated with unsatisfactory tumor control and long-term morbidity [28, 29]. In contrast, this might indicate a lower surgical risk profile of glioma located in the cerebral hemispheres and the posterior fossa, consistent to previous data, attesting glioma of the cerebral hemisphere and posterior fossa a comparatively superior PFS [5].

As this data emphasizes the impact of resection extent and residual tumor volume in pediatric low-grade glioma surgery, it subsequently highlights the importance of preoperative risk assessment and the role of technical modalities for intraoperative resection control including intraoperative ultrasound, high-field intraoperative MRI (iMRI) and electro-physiological intraoperative neuromonitoring (IOM), as some of these modalities have shown to play a crucial role in maximizing resection extent while preventing irreversible long-term neurologic impairment [30–32].

The underlying molecular mechanisms causing a tendency to growth deceleration of tumor residues still remain unclear. In a detailed in vitro analysis of oncogene-induced senescence (OIS) in a pilocytic astrocytoma model, Buhl et al. have demonstrated a significant reduction of growth by stimulation of pilocytic astrocytoma cells with rIL1B, while anti-inflammatory treatment with dexamethasone induced regrowth of senescent cells and inhibited the senescence-associated secretory phenotype (SASP) [21]. Based on the assumption of surgical intervention leading to the induction of local inflammatory processes in surrounding tissues, this may outline a possible explanation for indolent growth rates after radical resection, as cytokine concentrations and exposure to inflammatory processes may be considerably higher in smaller tumor residuals post extensive resection. Further investigation of molecular mechanisms of postoperative senescence in pLGG is warranted.

Comparison of postoperative TGV of BRAF V600E mutant LGG and BRAF wild-type LGG showed significantly higher postoperative tumor growth rates in BRAF V600E mutant LGG, which may suggest incompletely resected BRAF V600E positive pLGG as a high-risk subgroup, in line with previously published clinical data [17, 18].

Comparative analysis of pre- and postoperative tumor growth rates in BRAF-KIAA1549 (B-K) fusion positive and negative LGG showed no significant difference of mean TGV. B-K fusion was only detected in pilocytic astrocytoma and diffuse astrocytoma, consistent to a previously published screening of a large brain tumor cohort [33]. Notably, B-K fusion showed the highest frequency in supratentorial midline glioma excluding opticohypothalamic tumors (11/11, 100%), in line with previous publications [34]. Despite a limited overall conclusiveness of this data due to low case numbers, these key molecular alterations may play a significant role in terms of risk stratification and moreover represent a promising target for molecular therapies [16].

Moreover, the results of this study emphasize the distinct biology of pediatric versus adult LGG, as in a comparable study, Mandonnet et al. have shown no significant difference of growth rates before and after IR in adult WHO grade II glioma [35]. In consistence with the significantly higher tendency of adult LGG to evolve into higher-grade lesions, this may be considered as a consequence of fundamental molecular and genetic differences between these tumor entities [8]. As previously published by Pallud et al., the initial MRI growth rates of WHO II° gliomas in adult patients have shown long-term prognostic value [36].

The limitations of this study include the limited validity due to it’s retrospective approach. Moreover, overall conclusiveness of the data on the impact of BRAF alterations on postoperative TGV may be limited due to low case numbers included in the corresponding analyses. A significant number of cases were diagnosed before molecular testing for BRAF alterations was routinely performed, which resulted in a relatively high number of cases with unknown molecular BRAF aberration status in our cohort.

Regarding the method of tumor volume measurement and calculation of growth velocity it should be pointed out, that a reliable differentiation of residual tumor burden from unspecific postoperative signal intensity alterations in postoperative MRI scans can be very difficult, particularly in small potential postoperative residuals. To address this potential bias, only 3-month follow-up MRI examinations with contrast enhanced sequences were used as the earliest postoperative reference scan instead of immediate postoperative MRI data for a more reliable differentiation of residual tumor burden from unspecific postoperative signal intensity alterations. Moreover, central radiologic review reports of the Neuroradiology Reference Center of the German Society of Pediatric Oncology were consulted in all disputable cases. Patients were only included to the IR group in case of a confirmed tumor residual by the Neuroradiology Reference Center report. Nonetheless, a possible misinterpretation in distinct cases of very small tumor residuals cannot be excluded for sure, and might lead to an underestimated rate of achieved GTR and thus a relatively higher rate of achieved IR. Moreover, a possible misinterpretation in distinct cases of very small tumor residuals may possibly have an impact on calculated mean postoperative TGV, and may be—in contrast to a possible actual tumor regression—an alternative explanation for a slightly negative mean TGV after 2nd or 3rd IR (as illustrated in Fig. 2A, B).

## Conclusion

This study underscores the role of surgery within the treatment of pediatric LGG. Besides reduction of tumor mass effects to restore functional integrity of surrounding brain tissue and CSF circulation, the extent of surgery may impact the kinetics of pediatric low-grade glioma residuals post IR by inducing a significant deceleration of tumor growth, emphasizing the role of pursuing maximal possible tumor resection while preventing irreversible long-term neurologic impairment.

## Supplementary Information

Below is the link to the electronic supplementary material.

Supplementary file1 (PDF 439 kb)

## Data Availability

The datasets analyzed during the current study are available from the corresponding author on reasonable request.
