# Supplementary material for: Resection extent and BRAF V600E mutation status determine postoperative tumor growth velocity in pediatric low-grade glioma: results from a single-center cohort analysis
Source: J Neurooncol. 2022 Nov 1;160(3):567–76. doi: 10.1007/s11060-022-04176-4 (PMC9758245; doi:10.1007/s11060-022-04176-4)
Supplement: Supplementary file 1 — Supplementary file1 (PDF 439 kb) [file 11060_2022_4176_MOESM1_ESM.pdf]

	Low-grade glioma (LGG)							
	total	PA °1 [a]	GG °1 [b]	A °2 [c]	RGNT °1 [d]	SEGA °1 [e]	PXA °2 [f]	OGD °II [g]
**Sex**								
Male	95	66	20	7	1	1		
Female	96	71	16	7			1	1
**Age at diagnosis**				7.9 years (1 – 17)				
0 - 4	52	43	7	2				
5 - 9	55	44	6	4	1			
10 - 14	44	33	8	2		1		
15 - 17	40	17	15	6			1	1
**NF-1**	22	22						
**Localization**								
PF [h]	80	75	5	2	1			
SMG and OG [i]	55	49	2	4				
CH [j]	46	9	28	7			1	1
LV [k]	2			1		1		
Spinal cord	8	5						
**Resection extent**								
GTR [m]	65	38	18	8				1
STR [n]	58	42	10	3	1	1	1	
PR [o]	49	38	8	3				
No surgery	19	19						
**(Neo)adjuvant therapy**								
Chemotherapy	29	29						
Radiation	15	12		2			1	
Targeted therapy	5	5						
**total**	191	137	36	14	1	1	1	1

[a] Pilocytic astrocytoma °1
[b] Ganglioglioma °1
[c] Astrocytoma °2
[d] Rosette-forming glioneural tumor °1
[e] Subependymal giant cell astrocytoma °2
[f] Pleomorphic Xanthoastrocytoma °2
[g] Oligodendroglioma °2
[h] Posterior fossa
[i] Supratentorial midline glioma and Optic pathway glioma
[j] Cerebral hemisphere
[k] Lateral ventricle
[m] Gross-total resection
[n] Subtotal resection
[o] Partial resection

Supplemental table S1

Detailed demographic and clinical data of a single center cohort of 191 patients treated with LGG between 2006 and 2020.
